# Polymorphisms in reverse transcriptase and protease genes of HIV-1 subtype C from Mumbai

**DOI:** 10.1186/1471-2334-14-S3-E11

**Published:** 2014-05-27

**Authors:** Ritwik Dahake, Shraddha Mehta, Sneha Yadav, Abhay Chowdhary, Ranjana A Deshmukh

**Affiliations:** 1 Department of Virology, Haffkine Institute, Mumbai-400012, Maharashtra, India

## Background

Primary or transmitted HIV-1 drug resistance has caused an alarm in developed countries. While certain HIV-1 subtype C polymorphisms in relation to consensus subtype B sequences are known, there still exists a debate on the validity of these mutations being associated with/mistaken as, primary resistance. In this preliminary study, we have determined polymorphisms in reverse transcriptase (RT) and protease (PR) genes of HIV-1 subtype C from Mumbai, India.

## Methods

The study was performed using plasma samples from 24 antiretroviral therapy-experienced and drug-naïve patients employing a ‘home-brew’ semi-nested reverse-transcriptase-PCR followed by sequencing and sequence analysis. Analysis and interpretation of polymorphisms and other drug-resistance mutations was carried out using the Stanford HIV drug-resistance database. We also analysed Surveillance Drug Resistance Mutations (SDRMs) for PR gene.

## Results

We determined polymorphisms at a mutational frequency of 0.0670 ± 0.014 and 0.1337 ± 0.042, while 12.5% and 16.6% samples harboured drug-resistance mutations in RT and PR genes respectively. Substitutions greater than 50% were at positions D121, K122, T165, K166, K173, D177, T200, Q207, R211 for RT and L19, V82, M36, R41, L63, H69, L89, I93 for PR gene. Additionally, PR gene SDRMs were observed in 15.0% samples.

## Conclusion

Our study re-iterates that polymorphisms in HIV-1 subtype C from India may also include a number of major and minor/accessory mutations associated with resistance. We recommend that HIV subtype-specific drug-resistance databases be created to empower routine and unambiguous surveillance of drug resistance prior to initiating antiretroviral therapy, especially when including Protease Inhibitors.

